# High‐Spatiotemporal‐Resolution Ultrasound Flow Imaging to Determine Cerebrovascular Hemodynamics in Alzheimer's Disease Mice Model

**DOI:** 10.1002/advs.202302345

**Published:** 2023-11-14

**Authors:** Hsin Huang, Pei‐Ling Hsu, Sheng‐Feng Tsai, Yi‐Hsiang Chuang, De‐Quan Chen, Guo‐Xuan Xu, Chien Chen, Yu‐Min Kuo, Chih‐Chung Huang

**Affiliations:** ^1^ Department of Biomedical Engineering National Cheng Kung University Tainan 70101 Taiwan; ^2^ Department of Anatomy School of Medicine College of Medicine Kaohsiung Medical University Kaohsiung 80708 Taiwan; ^3^ Department of Medical Research Kaohsiung Medical University Hospital Kaohsiung 80708 Taiwan; ^4^ Drug Development and Value Creation Research Center Kaohsiung Medical University Kaohsiung 80708 Taiwan; ^5^ Department of Cell Biology and Anatomy College of Medicine National Cheng Kung University Tainan 70101 Taiwan; ^6^ Institute of Basic Medical Sciences College of Medicine National Cheng Kung University Tainan 70101 Taiwan; ^7^ Medical Device Innovation Center National Cheng Kung University Tainan 70101 Taiwan

**Keywords:** Alzheimer's disease, cerebrovascular hemodynamics, high‐frequency ultrasound, high‐resolution brain imaging, vector flow imaging

## Abstract

Although the relationships of cerebrovascular hemodynamic dysfunction with neurodegenerative diseases remain unclear, many studies have indicated that poor cerebral perfusion accelerates the progression of neurodegenerative diseases, such as Alzheimer's disease (AD). Small animal models are widely used in AD research. However, providing an imaging modality with a high spatiotemporal resolution and sufficiently large field of view to assess cerebrovascular hemodynamics in vivo remains a challenge. The present study proposes a novel technique for high‐spatiotemporal‐resolution vector micro‐Doppler imaging (HVμDI) based on contrast‐free ultrafast high frequency ultrasound imaging to visualize the cerebrovascular hemodynamics of the mouse, with a data acquisition time of 0.4 s, a minimal detectable vessel size of 38 µm, and a temporal resolution of 500 Hz. In vivo experiments are conducted on wild‐type and AD mice. Cerebrovascular hemodynamics are quantified using the cerebral vascular density, diameter, velocity, tortuosity, cortical flow pulsatility, and instant flow direction variations. Results reveal that AD significantly change the cerebrovascular hemodynamics. HVμDI offers new opportunities for in vivo analysis of cerebrovascular hemodynamics in neurodegenerative pathologies in preclinical animal research.

## Graphical abstract

High‐spatiotemporal‐resolution vector micro‐Doppler imaging (HVμDI) based on contrast‐free ultrafast high‐frequency ultrasound imaging is developed to visualize and quantify the cerebrovascular hemodynamics of the wild‐type (WT) and Alzheimer's disease (AD) mice. Irregular cerebrovascular hemodynamics including cerebral vascular density, diameter, velocity, tortuosity, cortical flow pulsatility, and instant flow direction variations in AD group are evaluated through the proposed novel technique.

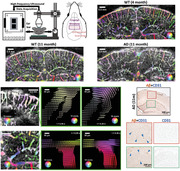

## 1 Introduction

Cerebral neurodegenerative diseases (CNDs) such as Alzheimer's disease (AD) are associated with brain vascular dysfunction and degeneration and may result in irreversible cognitive disorders.^[^ ^1^ ^]^ A major indicator of CNDs is the variation of the structure and function of brain vasculature, such the reduction in cerebrovascular density in AD mouse brain.^[^ ^2^ ^]^ In addition, some large population‐based studies reported that diminished cerebrovascular blood flow velocity preceded cognitive decline and hippocampal atrophy in AD.^[^ ^1^, ^3^ ^]^ Recent studies have suggested that changes in cerebrovascular hemodynamics alter the microcirculation and affect neurons.^[^ ^4^, ^5^ ^]^ For example, a decrease in blood flow pulsatility, one of the risk factors for cognitive decline, increases vascular resistance, lowers oxygen metabolism, and reduces the microcirculation, particularly in the cortical penetrating arterioles and ascending venules.^[^ ^4^, ^6^ ^]^ Furthermore, some studies have used optical imaging to measure the blood flow on the cortical surface of murine models, and results have indicated that cerebrovascular flow redistribution and reversal play vital roles in the development of cognitive disorders and cerebrovascular occlusion diseases.^[^ ^7^, ^8^ ^]^ Changes in the morphology and hemodynamics of cerebral vasculature have been demonstrated to be indicators of CNDs. However, achieving high‐spatiotemporal‐resolution imaging with deeper penetration for living animal cerebrovascular flow imaging remains a challenge.

Rodent models have been widely used in preclinical studies on CNDs. Brain angiograms including magnetic resonance imaging (MRI),^[^ ^9^ ^]^ positron emission tomography (PET),^[^ ^10^ ^]^ and optical imaging^[^ ^11^, ^12^ ^]^ have been used for in vivo cerebrovascular imaging of murine brains in CND research. However, the inadequate spatiotemporal resolutions of PET and MRI may limit their abilities to dynamically visualize cerebral microcirculation blood flow. Although optical imaging enables visualization of the microcirculation at the microscopic level (1–10 µm), only superficial cortical layers can be observed because of low light penetration. Moreover, medical ultrasound imaging has been applied for whole‐brain imaging in rodent models. For instance, functional ultrasound imaging (fUS) based on ultrafast power and color Doppler ultrasound imaging can be used to visualize brain activity at a high imaging frame rate (temporal resolution = 500 Hz).^[^ ^13^, ^14^ ^]^ However, the spatial resolution of these imaging techniques may be insufficient for mouse cerebrovascular imaging because of the limited operational ultrasound frequency (≈15 MHz). Moreover, the blood flow velocity measured through ultrafast color Doppler ultrasound imaging may be unstable as a result of uncertainty in Doppler angle estimation.^[^ ^15^ ^]^ Ultrasound localization microscopy (ULM) has been proposed for super‐resolution cerebral microcirculation imaging in small animals, which involves the injection of contrast agents (microbubbles) with resolutions of ≈9 and 20 µm for vessel size detection in 2D and 3D rodent cerebrovascular imaging, respectively.^[^ ^16^, ^17^ ^]^ Because ULM involves the tracking of microbubbles in the microcirculation, additional time (typically 100–150 s) is required for ultrasound data acquisition to accumulate sufficient microbubble paths for one cerebrovascular mapping, which reduces the temporal image resolution. In addition, the amount of the contrast agent injected and the duration for which the agent circulates must be controlled in ULM. Speckle decorrelation‐based approach to the cerebrovascular imaging of mice was also developed using 18.5 MHz ultrafast ultrasound imaging to achieve a favorable balance between the spatial (100 µm of the minimum detectable vessel size) and temporal (1 Hz) resolutions.^[^ ^18^ ^]^ However, this approach is not sensitive to slow flow signals. Therefore, high‐spatiotemporal‐resolution ultrasound cerebrovascular imaging that can extract stable hemodynamic information from rodent brains is still lacking for preclinical CND research.

A simple solution for increasing the spatial resolution of ultrasound imaging is the use of high‐frequency ultrasound (HFUS; >30 MHz) owing to its relatively short wavelength.^[^ ^19^ ^]^ HFUS has been used in multiple preclinical small‐animal imaging applications.^[^ ^20^, ^21^, ^22^ ^]^ Although commercially available HFUS machines can be used to achieve traditional Doppler imaging, their spatial resolutions are insufficient for rodent cerebrovascular imaging. Fortunately, ultrafast HFUS imaging, specifically HFUS micro‐Doppler imaging (HFμDI), has been proposed to increase the Doppler imaging resolution for microcirculation imaging in small animals.^[^ ^23^, ^24^ ^]^ Because the backscattering signals from erythrocytes increase with the operational ultrasound frequency,^[^ ^25^ ^]^ the microcirculation signals can be clearly extracted using HFUS without the use of microbubbles. A singular value decomposition (SVD) filter is frequently used to extract blood flow signals; however, the additional background noise and intensity fluctuation leads to low vessel visibility, which in turn reduces the imaging contrast.^[^ ^26^ ^]^ Therefore, to enhance the cerebral vasculature, methods to remove the background noise must be identified.

The accurate measurement of flow velocity is another challenge in brain Doppler imaging because the complicated tortuosity of cerebral vasculature makes the Doppler angle difficult to define. ULM enables the accurate measurement of flow velocity in the brain by tracking the microbubbles in the vasculature^[^ ^16^, ^17^ ^]^; however, its temporal resolution is low, which hinders the dynamic measurement of blood flow. The temporal resolution of the speckle decorrelation‐based approach (1 Hz,^[^ ^18^ ^]^) was also inadequate for mice brain velocimetry. Use of vector flow imaging based on speckle tracking approach is one solution for providing dynamic blood flow information with a very high temporal resolution.^[^ ^27^ ^]^ However, no study has reported the use of vector flow imaging in rodent brains because speckle tracking is highly dependent on image quality, and speckle patterns obtained from erythrocytes, particularly in small vessels, are blurry. In other words, the use of HFUS vector flow imaging based on multiple tilted plane waves exhibits a high potential for providing dynamic cerebrovascular information in the rodent brain.

In the present study, we proposed a novel high‐spatiotemporal‐resolution vector micro‐Doppler imaging (HVμDI) technique based on ultrafast 40‐MHz HFUS imaging to visualize the mouse cerebral vasculature without the injection of microbubbles. Morphology‐based enhancement algorithm was utilized to improve the cerebrovascular visibility and vector flow imaging based on an adaptive filter was proposed for estimating the details of cerebrovascular flows. The efficacy of the proposed imaging method was verified using a flow phantom. In vivo, animal experiments were carried out on young (4 months) wild‐type (WT), aged (11 months) WT, and AD (triple‐transgenic, 3xTg; 11 months) mice. Since the imaging resolution of the current state‐of‐the‐art commercial high‐frequency ultrasound system is still insufficient for mice brain cerebrovascular imaging, we hypothesized that the hemodynamic variations including cerebral vascular density, vascular diameter, flow velocity, vascular tortuosity, cortical flow pulsatility, and instant flow direction across different mice brain regions between healthy and disease groups can be observed and quantified by the proposed method yielded high‐spatiotemporal‐resolution cerebrovascular images. Thus, this method has the potential to serve as a new tool in CND rodent model research.

## 2 Results

### 2.1 Experimental Illustration and Implementation of HVμDI

**Figure 1:**
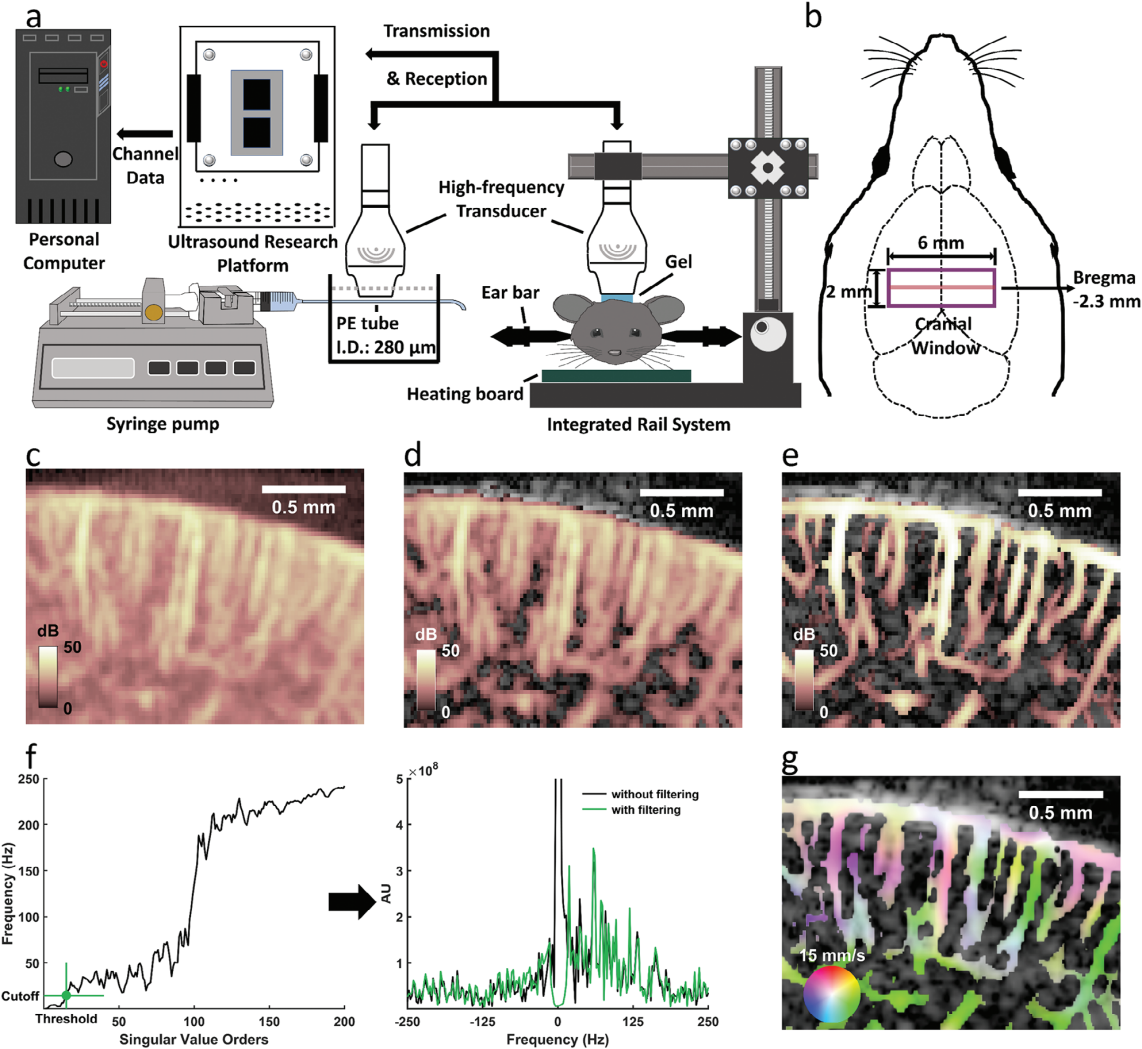
Schematic of the experimental design and working principle of HVμDI. a) Experimental setup for phantom and animal studies. b) Mouse brain scanning position: A cranial window was surgically created between the bregma and lambda for ultrasound imaging, and the ultrasound transducer was positioned at the bregma at −2.3 mm. c) Typical HFUS cerebrovascular image obtained from the cortical region of a young WT mouse after SVD filter: Although the erythrocyte signals were preliminarily extracted, background noise remained in the image. d) Cerebrovascular image obtained after the application of BH transform: BH transform was applied to eliminate the background noise in order to enable a clearer visualization of the vascular region. e) Cerebrovascular image obtained after VE: VE was finally applied to penalize the background noise after BH transform and enable enhanced visualization of the morphological features of the vasculature. f) Adaptatively determined cutoff frequency and the frequency spectrum of the high‐pass filter: The cutoff frequency was adaptatively selected from the frequency curve in SVD filter. High‐pass filter was then performed using this cutoff frequency on the reshaped IQ data to eliminate unwanted noise. g) HFUS vector flow image and 360° color map representing both flow velocity and direction.

A schematic of the experimental design is shown in **Figure** **1**a. Mice were anesthetized through the intraperitoneal injection of pentobarbital (1 g kg^−1^). Ear bars were used to secure the heads of the mice to the stereotaxic frame. A cranial window was surgically created between the bregma and lambda for ultrasound imaging. The HFUS array transducer was positioned at the bregma −2.3 mm, as shown in Figure 1b. A straight microtube flow phantom containing blood‐mimicking fluid was used to evaluate the efficacy of the proposed imaging method; the flow velocities were preset as 1.8, 3.6, 5.4, 7.2, 9.0, 10.8, 12.6, and 14.4 mm s^−1^.

An SVD filter was first applied to the compounded ultrasound in‐phase and quadrature (IQ) data to extract the cerebral vasculature signals. Figure 1c shows a typical vascular image of the cerebral cortical region obtained from a young WT mouse after an SVD filter was applied to the IQ data. Although the cerebral vasculature in this image can be roughly distinguished, the additional background noise in the image limits the visibility. To suppress this background noise, bowler‐hat (BH) transform was applied (Figure 1d). However, some residual random noise caused by the morphological features remained. Therefore, a blood vessel enhancement (VE) algorithm based on the Hessian matrix was applied to penalize the background noise and achieve considerable VE and thus enable clear visualization of the cerebral vasculature (Figure 1e). For vector‐flow processing, a thresholding frequency was automatically and adaptively determined on the basis of the frequency curve obtained after SVD filtering (Figure 1f, left). This thresholding frequency was considered as the cutoff frequency for temporal high‐pass filtering (Figure 1f, right) of the reshaped 4D IQ data. The vector velocities were then estimated in every voxel in the filtered 4D IQ data by using the multibeam Doppler strategy. Finally, HVμDI was performed to obtain high‐spatiotemporal‐resolution information regarding mouse cerebral vasculature, including the blood flow direction and velocity (Figure 1g).

### 2.2 Flow Velocity Estimation with Adaptive Filters In Vitro

**Figure 2:**
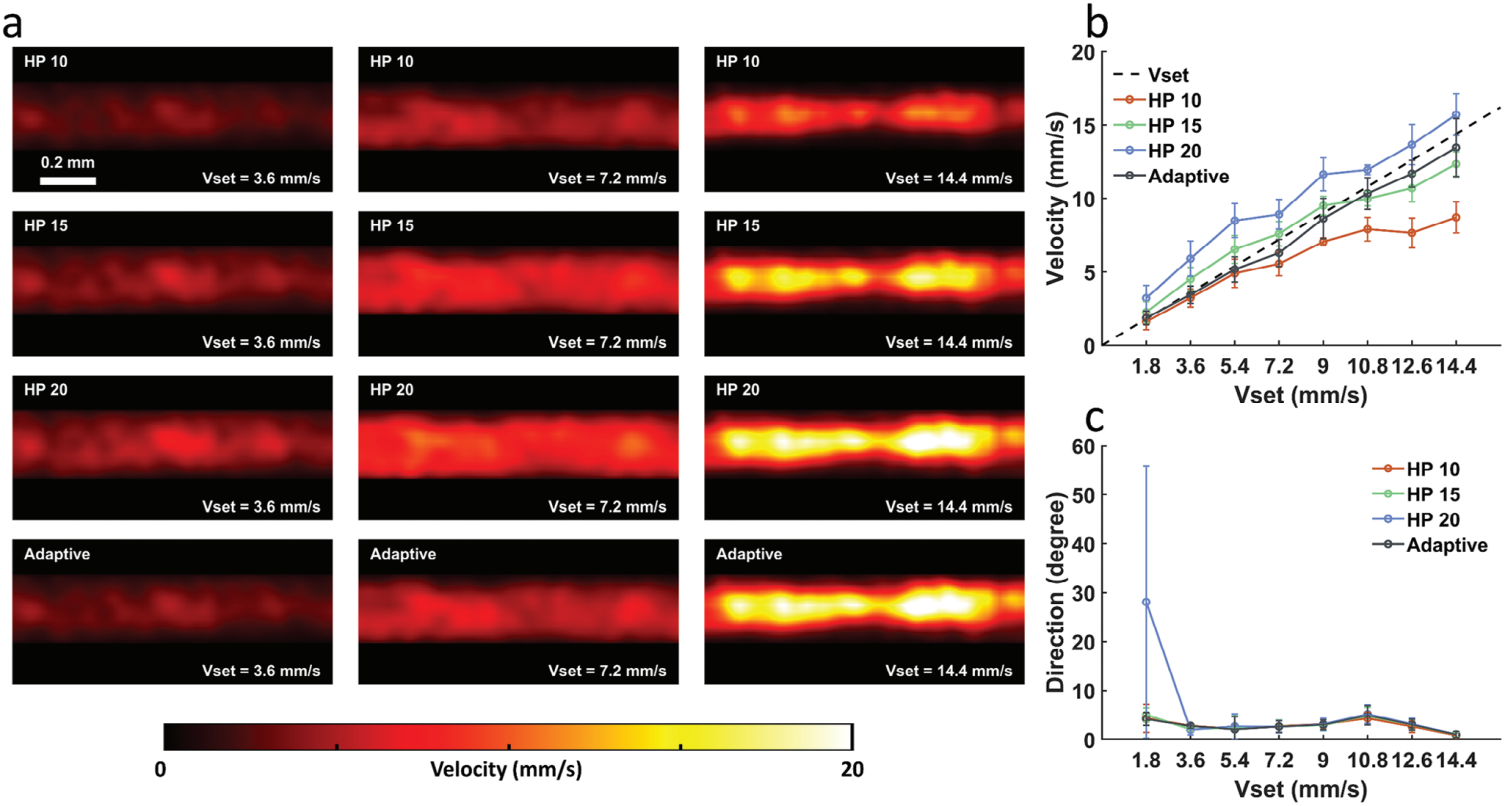
Flow phantom validation for HVμDI. a) Flow velocity maps obtained using the proposed method with adaptive cutoff frequencies as well as fixed cutoff frequencies ranging from 10 to 20 Hz at multiple preset flow velocities (*V*_set_): The velocity at a fixed frequency of 20 Hz was overestimated when the preset velocity was low (3.6 mm s^−1^), whereas the velocities at fixed frequencies of 10 and 15 Hz were underestimated when the preset velocity was high (14.4 mm s^−1^). The velocities estimated using the proposed strategy were all close to the three preset velocities. b) Quantified comparisons of flow velocities. The velocity measured using the proposed strategy (black line) was closer to the preset velocity (black dashed line) than were those measured using other methods. c) Quantified comparisons of flow directions: The flow directions were generally stable for all the cutoff frequencies except for the fixed cutoff frequency of 20 Hz at a low present velocity of 1.8 mm s^−1^. The error bars denote standard deviations from four individual measurements. HP represents high‐pass.

Setting an appropriate cutoff frequency is crucial for flow velocity estimation. The flow phantom experiment was performed to confirm the suitability of the proposed SVD‐based adaptive filter method. The flow velocities measured in the straight microtube flow phantom are shown in **Figure** **2**. Estimated velocities with fixed cutoff high‐pass frequencies of 10, 15, and 20 Hz are also presented for comparison. Figure 2a presents the measured flow velocity maps drawn at preset flow velocities of 3.6, 7.2, and 14.4 mm s^−1^. The velocity maps based on flow velocities measured in the straight microtube varied with the cutoff frequency of the high‐pass filter. For example, the velocities at cutoff frequencies of 10 and 15 Hz were underestimated when the preset velocity was 14.4 mm s^−1^, and that at a cutoff frequency of 20 Hz was overestimated when the preset velocity was 3.6 mm s^−1^. A comparison of all the estimated velocities and the preset velocities is presented in Figure 2b, where the error bars represent the standard deviations from four individual measurements. The velocity measured using from the proposed method (black line) was closer to the preset values (black dashed line) than were those measured using other methods; this outcome indicated the accuracy of the proposed method in estimating velocities compared with the fixed cutoff frequency approach. Most errors of the proposed method were <10% (one for 11.1%), whereas the errors of others were generally larger (even exceeded 50%). Figure 2c illustrates the estimated flow directions in the microtube. These flow directions were mostly consistent regardless of the cutoff frequency settings, except for a cutoff frequency of 20 Hz at a low preset velocity of 1.8 mm s^−1^ because a higher cutoff frequency can easily affect slower flow signals, which in turn can lead to phased distortion. In other words, the proposed filtering strategy enabled the stable estimation of both flow velocity and direction because the optimal cutoff frequency could be determined adaptively.

### 2.3 Visualization and Quantification of the Cerebral Vasculature of Living Mice Through HVμDI

**Figure 3:**
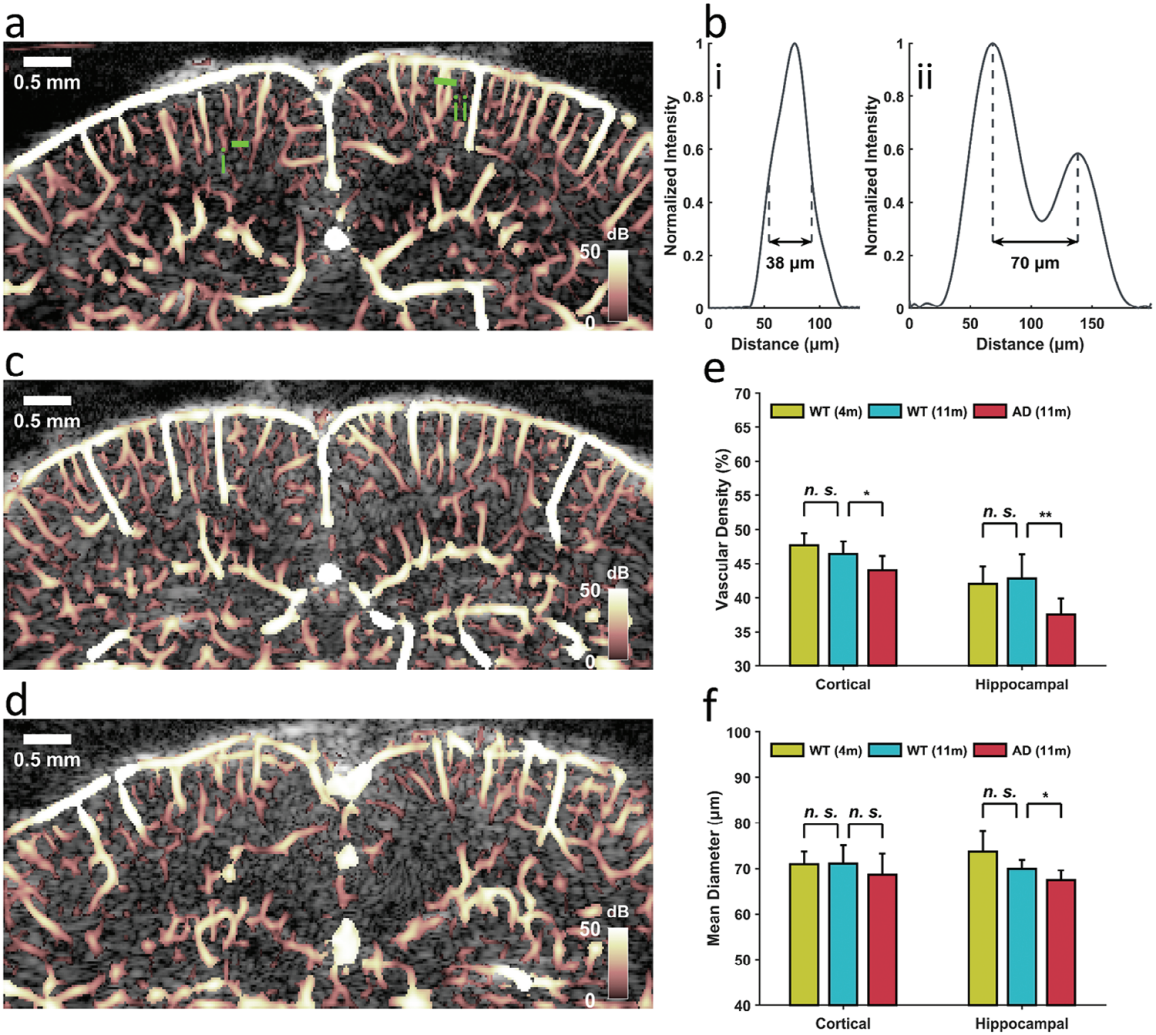
Cerebral vasculature imaging of living mouse brains through HVμDI. a) Cerebrovascular image from a young WT mouse: The vasculature could be clearly visualized after SVD filter and morphological enhancement (BH transform and VE). b) Two cross‐sectional profiles (i and ii) of vessels from the selected section in a: The minimal detectable vessel diameter was 38 µm, and the minimal resolvable distance between two vessels was 70 µm. c,d) Cerebrovascular images from an aged WT (c) and an AD mouse (d): Although the vasculature was similar overall between the young and aged WT mice, the vasculature of the AD mice was evidently more tortuous and blurred. e,f) Quantification of the vascular density (e) and mean vessel diameter (f) in the cortical and hippocampal regions) respectively, in all the groups: Significant decreases in vascular density were found between the aged WT and AD groups in both the cortical (*p* <0.05) and hippocampal (*p* <0.01) regions. Regarding the mean vessel diameter, a significant decrease was found only in the hippocampal region in both the aged WT and AD groups (*p* <0.05). Data are presented as means ± standard deviations. *p*‐values are calculate using one‐way analysis of variance, ^*^ *p* <0.05, ^**^ *p* <0.01. Sample size: 8 for WT (4m), 8 for WT (11m) and 7 for AD (11m).

**Figure 4:**
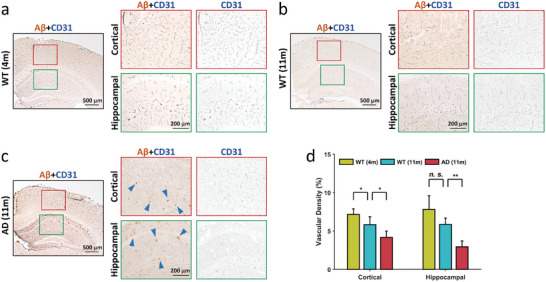
Histological analysis through double staining immunohistochemistry of CD31 and Aβ. a–c) The histological images obtained from young WT (a), aged WT (b), and AD (c) mice. The zoomed‐in images in cortical and hippocampal regions (red and green windows in each figure, respectively) were presented for comparisons, respectively. The deposition of Aβ peptides were observed in both cortical and hippocampal regions of AD group (Aβ+CD31 images in Figure 4c; blue triangles indicate the deposition). The dark lines in the CD31 images indicate the vessels, and the vascular density decreased in AD group (CD31 images in (c). d) Quantification of the vascular density through histological analysis in the cortical and hippocampal regions, respectively, from all groups. Significant decreases in vascular density were found between the aged WT and AD groups in both the cortical (*p* <0.05) and hippocampal (*p* <0.01) regions. Data are presented as means ± standard deviations. *p*‐values are calculate using one‐way analysis of variance, ^*^ *p* <0.05, ^**^ *p* <0.01. Sample size: 5 for WT (4m), 5 for WT (11m), and 5 for AD (11m).

**Figure** **3**a presents a typical cerebrovascular image obtained from a young WT mouse through HVμDI (the colors represent the backscattering signal intensity). Both the cortical and hippocampal vascular structures were visualized in detail without the use of an ultrasonic contrast agent. HVμDI can directly detect the intrinsic contrast of erythrocytes with a considerably shorter acquisition time (0.4 s) than that required by ULM (typically > 100 s^[^ ^16^, ^17^ ^]^). The minimal detectable vessel size was 38 µm, and the minimal resolvable distance between two detectable vessels was 70 µm, as can be seen in the cross‐sectional profiles of the vessels (Figure 3b). These resolutions are superior to those of other contrast‐free ultrasound modalities.^[^ ^13^, ^14^, ^18^ ^]^ The difference in cerebral vasculature between the young WT mice (Figure 3a) and aged WT mice (Figure 3c) was not obvious, whereas the vasculature in the AD mice was more blurred and tortuous (Figure 3d). In addition, the structural difference within the brain was clearly observed (Figure 3d), it was associated with the accumulation and aggregation of amyloid‐β (Aβ) in brain parenchyma, which is a critical pathological feature of AD.^[^ ^1^ ^]^ Such structural difference was also observed between young and aged WT mice (Figure 3a,c), it may be related to the fact that aging is one of the primary risk factors of neurodegeneration.^[^ ^28^ ^]^ Figure 3e presents the vascular density in the both cortical and hippocampal regions in all three groups; no significant differences in vascular density were noted between the young and aged WT mice across regions; however, a significant reduction in vascular density was observed in the AD group compared with the aged WT group (cortical: −5%, *p* <0.05; hippocampal: −12%, *p* <0.01). These results are in agreement with the findings of a mouse study obtained through optical microscopy.^[^ ^29^ ^]^ Figure 3f presents a comparison of the quantitative measurements of mean vessel diameter at the cortical and hippocampal regions between the groups. The vessel diameter in the hippocampal region was significantly decreased in the AD group compared with the aged WT mice (−3 µm, *p* <0.05); however, the difference in the vessel diameter in the cortical region between the groups was not significant. These results suggest that AD alters the cerebral vasculature. Double staining immunohistochemistry of cluster of differentiation 31 (CD31) and Aβ was utilized to determine the vascular changes and Aβ deposits in the cortical and hippocampal regions of mice. Aβ peptide deposition was evident in both the cortical and hippocampal regions of the AD group (Aβ+CD31 images in **Figure** **4**c; blue triangles indicate the deposition), whereas it was absent in the aged WT mice (Figure 4a). The CD31‐immunoreactive signals (Figure 4a–c; lines in the figures indicate the vessels) that were isolated from the double stain revealed a reduction in capillaries within both the cortical and hippocampal regions of AD group (CD31 images in Figure 4c). The vascular density was then quantified by using ImageJ software (Figure 4d), and vascular density was decreased significantly (cortical: −25%, *p* <0.05) for the aged WT group compared with the young WT group; and for the AD group compared with the aged WT group (cortical: −50%, *p* <0.05; hippocampal: −29%, *p* <0.01). These findings verified alterations in vascular density (Figure 3) resulting from Aβ peptide deposition. However, while histological analysis stands as a prominent method for investigating cerebral neurodegenerative diseases in rodent models, it requires the sacrifice of animals and does not provide insights into the hemodynamics of the brain.

### 2.4 Visualization and Quantification of Cerebrovascular Hemodynamics Through HVμDI

**Figure 5:**
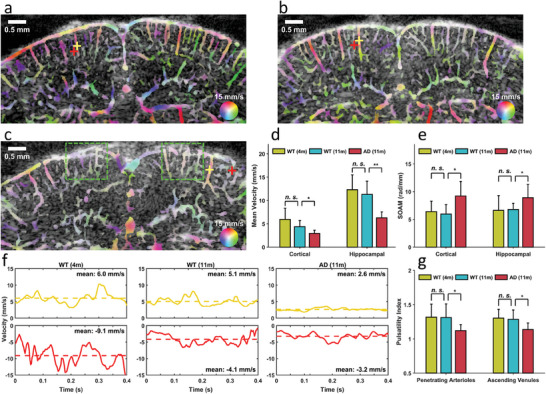
Cerebrovascular hemodynamics of living mouse brains observed through HVµDI. a–c) Cerebral vector flow images obtained from young WT (a), aged WT (b), and AD (c) mice: The 360° color map represents both flow velocity and direction. Flow velocity slightly decreased in the young and aged WT groups but considerably decreased in the AD group. For example, the flow velocities decreased in the cortical regions of the AD mice (two green dashed line windows in c). d,e) Quantification of the mean velocity (d) and SOAM (e) of the cerebral vasculature: Mean velocity represents time‐averaged flow velocity, and a higher SOAM represents higher tortuosity. Significant differences in both the parameters were observed between the aged WT and AD mice (*p* <0.05); however, the mean velocity decreased more significantly in the hippocampal region (*p* <0.01) than in the cortical region. f) Temporal profiles of the flow velocities in the penetrating arterioles and ascending venules, measured at cross label locations (a–c) (red indicates the locations in the penetrating arterioles, and yellow indicates the locations in the ascending venules): The dashed lines in each profile indicate the mean flow velocity. A negative value indicates that the blood flowed from top to bottom (penetrating), and a positive value indicates that the blood flowed from bottom to top (ascending). The flow profiles of the young and aged WT groups exhibited high pulsatility, whereas those of the AD group exhibited low pulsatility. g) Quantification of pulsatility in all groups: A lower pulsatility index indicates lower pulsatility. Significant decreases in pulsatility were found in both the penetrating arterioles and ascending venules in the AD group (*p* < 0.05). Data are presented as means ± standard deviations. *p*‐values are calculate using one‐way analysis of variance, ^*^ *p* <0.05, ^**^ *p* <0.01. Sample size: 8 for WT (4m), 8 for WT (11m), and 7 for AD (11m).

**Figure** **5**a presents a typical cerebral vector flow image obtained from a young WT mouse through HVμDI. The 360° color map based on the HSV color space represents both flow velocity and direction. For example, the green color indicates blood flow from left to right, with the color saturation representing the magnitude (i.e., flow velocity). The major advantage of the HVμDI approach is that the flow information in every voxel (i.e., 2D spatial and temporal) can be determined, which resulted in the efficient visualization of cerebrovascular hemodynamics (Figure 5a; Video S1, Supporting Information). Cerebral vector flow images for the aged WT mice (Figure 5b; Video S2, Supporting Information) and AD mice (Figure 5c; Video S3, Supporting Information) are also presented for visual comparison. The overall flow velocity was slightly lower in the aged WT mice than in the young WT mice; however, the flow velocity was considerably lower in the cortical region of the AD mice (green dashed line windows in Figure 5c), and the reduction in flow velocity in the AD mice was evident. Furthermore, the mean velocities (i.e., time‐averaged flow velocities) were quantified (Figure 5d); the results revealed no significant difference in the mean velocity in either the cortical or hippocampal region between the young and aged WT mouse groups; however, the mean velocity in the AD mice was significantly reduced d compared with that in the aged WT mice (cortical: −34%, *p* <0.05; hippocampal: −45%, *p* <0.01), and this finding was consistent with the AD pathology.^[^ ^1^, ^3^ ^]^ Cerebrovascular tortuosity was evaluated using the sum of angle metric (SOAM),^[^ ^30^, ^31^ ^]^ which was derived from vector flow information. Mean velocities and tortuosity are primarily used to quantify cerebral flow in the resting state.^[^ ^32^ ^]^ A higher SOAM value indicates higher tortuosity. No significant differences in SOAM values were found between the young and aged WT mice (Figure 5e); however, the SOAM values of the AD mice were significantly increased in both the cortical (+55%, *p* <0.05) and hippocampal (+31%, *p* <0.05) regions compared with the aged WT mice. This result is consistent with a previous finding, namely that the deposition of Aβ peptides, a major contributor of AD, primarily increases the tortuosity of the cerebral vasculature, particularly in the cortical region.^[^ ^33^, ^34^ ^]^ The high temporal resolution (500 Hz) further enabled the evaluation of hemodynamic changes in the temporal domain. Figure 5f presents the dynamic flow velocities measured in the cortical penetrating arterioles (red color) and ascending venules (yellow color) for each mouse group (the measured locations are marked in yellow and red in Figure 5a–c). The dashed lines in the figures represent the mean velocity, and the negative (downstream) and positive (upstream) values of velocity indicate the flow directions. Pulsatile flow patterns were observed in both penetrating arterioles and ascending venules in the young and aged WT mice but not in the AD mice. Several studies have reported that large cerebral vessels such as middle cerebral arteries and surface veins are pulsatile and play a crucial role in brain microcirculation.^[^ ^35^, ^36^ ^]^ HVμDI can directly assess the pulsatility of microcirculation in small cortical regions owing to its high spatial resolution. Pulsatility was quantified using the pulsatility index.^[^ ^37^ ^]^ No significant difference in the pulsatility index was observed between the young and aged WT groups for either vessel (Figure 5g); however, a significant decrease in the pulsatility index was observed between the aged WT and AD groups for both vessels (−15% in penetrating arterioles and −12% in ascending venules, both *p* <0.05), indicating a lower efficiency of microcirculation in the cortical region of the AD brain.^[^ ^4^ ^]^

### 2.5 Visualization and Quantification of the Directionality of Cerebrovascular Flow Through HVμDI

**Figure 6:**
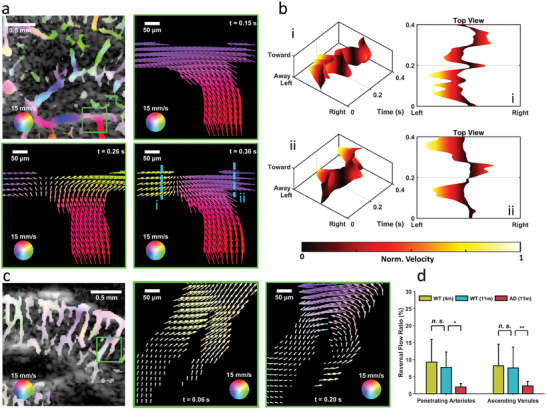
Instantaneous changes in cerebrovascular flow in mouse brains. a) Typical image of cerebral vector flow in the hippocampal region of a young WT mouse: The zoomed‐in (green window) vector flow images at multiple temporal instants from 0.15 to 0.36 s are shown. The color of arrows based on a 360° color map represents both flow velocity and direction. The length of the arrows represents the relative magnitude of velocity. The flow directions are not always unidirectional with time, even within the same vessel. b) Flow direction profiles of the two selected cross sections (i and ii) in a (left–right and toward–away of transducer, as functions of time) and their corresponding top view plots: The directions of blood flow in the left (i) and right (ii) branches apparently reversed several times in the horizontal direction. c) Typical vector flow image of the cortical region of an AD mouse and zoomed‐in (green window) vector flow image at multiple temporal instants from 0.06 and 0.20 s (the scale of the arrow length is three times that of the one in a for more favorable visualization): The blood flow direction in the left branch reversed to the vertical direction. d) Quantification of the reversal flow ratio: The reversal flow ratio denotes the percentage of reversal flow in all the recognized vascular regions. Significant decreases in the reversal flow ratio were found in both the penetrating arterioles and ascending venules in the AD group compared with the aged WT group (*p* <0.05). Data are presented as means ± standard deviations. *p*‐values are calculate using one‐way analysis of variance, ^*^ *p* <0.05. Sample size: 8 for WT (4m), 8 for WT (11m), and 7 for AD (11m).

**Figure** **6**a (Video S4, Supporting Information) presents a typical image of cerebral vector flow in the hippocampal region of a young WT mouse. The zoomed‐in regions (green window) at multiple temporal instants are plotted from 0.15 to 0.36 s. The arrows and colors represent the flow direction and magnitude. Evidently, the blood flow was from the bottom and right branches to the left branch at 0.15 s. At 0.26 s, a part of the bottom flow was separated to the right branches. Then, at 0.36 s, the right, left, and bottom flows were turned toward the middle. Because the middle part had no outflow, another vessel should have been present in the other imaging plane because cerebral vasculature is inherently a 3D structure. The flow profiles of two selected cross sections (labeled in Figure 6a, zoomed‐in region at a temporal instant of 0.36 s) were plotted to achieve more favorable visualization of the flow pattern as a function of time (Figure 6b). The blood flow direction in the left (section i) and right (section ii) branches apparently reversed in the horizontal direction, as clearly visualized in their corresponding top views. However, the bottom flow remained in the ascending direction. In addition to the horizontal reversal flow, a vertical reversal flow was observed in some vertical vessels. Figure 6c presents an example of reversal flow in the vertical direction in an AD mouse and corresponding zoomed‐in images (Video S5, Supporting Information). Ascending flows were observed in both (left and right) branches at a temporal instant of 0.06 s. However, the flow directions of the left branch and mainstream were reversed in a vertical direction at a temporal instant of 0.20 s. We calculated the reversal flow ratio to quantify the percentage of the reversal flow in the recognized vessels (Figure 6d). No significant difference in the reversal flow ratio was noted in either the penetrating arterioles or ascending venules between the young and aged WT groups; however, the reversal flow ratio was significantly reduced in the AD group compared with the aged WT group (−74% in penetrating arterioles, *p* <0.01; −70% in ascending venules, *p* <0.05), indicating that AD altered the cerebrovascular perfusion efficiency. All these results indicate the ability of HVμDI to accurately map dynamic cerebrovascular flow directionality.

## 3 Discussion

Cerebrovascular hemodynamics play a crucial role in the pathogenesis of AD, and changes in cerebrovascular hemodynamics result in Aβ accumulation in the brain, which further leads to reduced cerebral blood flow.^[^ ^38^ ^]^ Therefore, a tool that can be used to evaluate the cerebrovascular hemodynamics in small cerebral vessels becomes essential for AD research. In the present study, HVμDI, a novel ultrasound imaging technique, was proposed to measure the cerebrovascular morphology and hemodynamics, particularly flow direction and velocity, in a mouse model with high temporal (500 Hz) and spatial (minimal detectable vessel size, 38 µm) resolutions through vector flow technology. Using the ultrasound Vantage 256 system with a high‐frequency (40 MHz) array transducer provides a high‐spatiotemporal‐resolution imaging and thereby ensures the capability for evaluating the cerebrovascular hemodynamics of small cerebral vessels, which is essential since the cerebrovascular hemodynamic changes in small cerebral vessels easily affect the microcirculation and neurons in AD research.^[^ ^4^, ^5^ ^]^ To extract the blood signals from the cerebral vasculature, SVD filtering has been widely used in fUS and ULM to enhance the resolution of vasculature in organs.^[^ ^14^, ^17^ ^]^ In the most vector flow imaging, a high‐pass filter with a fixed cutoff frequency can be applied to extract blood signals.^[^ ^39^, ^40^ ^]^ By contrast, in the present study, we proposed an adaptive filtering strategy that to find the cutoff frequency via SVD filter and then use a high‐pass filter to extract blood flow signals based on this cutoff frequency. The cutoff frequency was derived from the *T*_L_th singular vector in the *V* temporal matrix (both from SVD) and was considered to be the threshold based on both spatial and temporal characteristics.^[^ ^41^ ^]^ The proposed technique considerably improved the accuracy of the estimated flow velocity in phantom experiments (mostly under 10%) compared with the fixed cutoff frequency approach (Figure 2).

In the present study, we applied a morphology‐based strategy that involved a hybrid of BH transform and Hessian‐based VE to enhance the cerebral vasculature and to suppress background noise after using a high‐pass filter to extract backscattering signals from erythrocytes. The minimal detectable vessel diameter was 38 µm, and the minimal resolvable distance was 70 µm, both of which were more favorable than the corresponding values reported by a previous murine study that applied HFUS alone for mouse cerebrovascular imaging.^[^ ^24^ ^]^ Moreover, in the present study, the cerebral vascular density was significantly reduced in the AD mice (−5% in the cortical region, −12% in the hippocampal region; Figure 3e), which was consistent with the finding of a previous study^[^ ^29^ ^]^; however, that previous study used microscopy for the ex vivo estimation of vascular density in AD mice. In addition to the vascular density, the vessel diameter was significantly reduced in the hippocampal regions of the AD mice in the present study (Figure 3f); this finding was consistent with the result of a previous study that used optical tomography for ex vivo imaging of murine brains with AD.^[^ ^42^ ^]^ Several reports have suggested that even slight changes in cerebral vessel diameters may result in irregular cerebrovascular resistance and an irregular flow rate,^[^ ^43^, ^44^ ^]^ which in turn can lead to insufficient brain perfusion and Aβ accumulation and eventually cause dysregulation of normal metabolic function and consequently the development of AD.^[^ ^1^, ^3^, ^38^ ^]^

In addition to the analysis of the morphological structure of cerebral vasculature, the flow velocity was measured in mouse brains because cerebrovascular dysregulation can be considered an early marker of cognitive decline before changes in AD biomarkers can be detected.^[^ ^1^, ^3^ ^]^ Evidently, in our AD mice, the cerebrovascular mean velocity was significantly reduced (−34% in the cortical region, −45% in the hippocampal region; Figure 5d); this finding can be attributed to the dysregulation of cerebrovascular function such as reductions in vascular density and vessel size. Cerebrovascular tortuosity is another biomarker of AD^[^ ^34^, ^45^ ^]^ and can be obtained using the SOAM derived from the proposed HVμDI in AD mice (+55% and +31% in the cortical and hippocampal regions, respectively; Figure 5e). The SOAM has been used to represent the geometrical properties of blood vessels.^[^ ^30^ ^]^ A recent study used ULM to determine the tortuosity of aging mouse brains.^[^ ^46^ ^]^ ULM employs the optimal pairing function to identify the trajectories of microbubbles. However, sufficient microbubble accumulation takes a long time, which consequently increases the signal acquisition time. By contrast, the proposed HVμDI needs only short acquisition time (< 1 s) without injecting contrast agent because the backscattering signals come from erythrocytes directly.

An increase in the stiffness of cerebral vasculature reduces the pulsatility of blood flow.^[^ ^31^ ^]^ In the present study, the pulsatility was reduced in the AD mice in both the penetrating arterioles and ascending venules (Figure 5g); this finding was consistent with the pathogenesis of AD, namely that Aβ accumulation leads to cerebrovascular arteriosclerosis.^[^ ^38^ ^]^ Due to the high spatial (minimal detectable vessel size, 38 µm) and temporal (500 Hz) resolutions of HVμDI, the present work determines the pulsatility of a single vessel in the cortical regions (including penetrating arterioles and ascending venules), which becomes even more essential for AD research since these cortical vessels are responsible for the exchange of most gases, metabolites, and nutrients in brain.^[^ ^4^, ^6^ ^]^ Recently, functional ULM was proposed for the quantification of cerebrovascular hemodynamics at a spatial resolution of 6.5 µm and a temporal resolution of 1 Hz;^[^ ^47^ ^]^ however, determining the pulsatility at such temporal resolutions was still difficult, and the data acquisition time was longer than that of conventional ULM. In addition, fUS also enables the detection of cerebrovascular flow with high temporal resolution of 500 Hz; however, its spatial resolution is only ≈100 µm,^[^ ^14^ ^]^ which is insufficient to resolve the hemodynamic changes in the small cortical vessels. A recent study reported a decrease in cortical pulsatility in AD mice through fluorescence imaging;^[^ ^48^ ^]^ however, owing to the highlight attenuation in fluorescence imaging, a scanning depth from the surface of ≈250 µm was used. By contrast, the field of view in HVμDI was sufficient to visualize the deeper hippocampal regions of mouse brains.

Cerebral vasculature images from a young WT mouse through commercial high‐frequency ultrasound imaging system (Vevo 2100, FujiFilm, VisualSonics, Toronto, ON, Canada) were presented as a control group in Figure S1. The operational frequency was set to 40 MHz and “slow flow” mode was selected for the cerebrovascular image. It is obvious that commercial system only can detect the blood flows in few larger vessels of the mice brain (Figure S1a, Supporting Information). Figure. S1b,c (Supporting Information) further demonstrated the zoomed‐in images in cortical and hippocampal regions (red and green windows in Figure S1a, Supporting Information), respectively. Again, there were few flow signals were detected in the cortical region (Figure S1b, Supporting Information), and only larger cerebral flows in hippocampal region were obtained (Figure S1c, Supporting Information). Besides, the commercial system cannot provide the vector flow information. This result also showed that the proposed HVμDI exhibits an excellent imaging resolution in cerebral vasculature imaging compared to the state‐of‐the‐art commercial system. Furthermore, commercial system only provides the directional information (toward or away from transducer [red or blue colors in Figure S1, Supporting Information]); while the HVμDI provides the vector flow information (Figure 5a–c). Therefore, it remains difficult to recognize the cerebral vasculature and evaluate the cerebral hemodynamics through commercial system.

A notable finding in the cerebral vasculature was the reversal flow dynamics (Figure 6). Unlike most arteries and veins, which have a unidirectional flow, the cerebrovascular blood flow direction appears to change over time in both the horizontal and vertical directions even within a single vessel (Figure 6a,c). This finding is in agreement with that of a recent report that observed reversal flow in the cerebral capillaries of WT mice through optical microscopy.^[^ ^49^ ^]^ This phenomenon may be attributable to the complex network of the cerebral vasculature with extensive redundant connections as well as the blood flow communication network being related to brain activity.^[^ ^7^, ^50^, ^51^ ^]^ The decrease in the reversal flow ratio in AD mice may imply a reduced ability of the blood flow network to communicate with the brain and therefore lower microcirculation efficiency, which is a common sign of AD.^[^ ^1^, ^3^ ^]^ However, few studies have investigated this phenomenon, and thus further evidence is required. Nevertheless, the proposed HVμDI paves a new way for the investigation of cerebrovascular hemodynamics in small animal pre‐clinical studies.

There are some limitations of the proposed HVμDI. Since the minimal detectable vessel diameter was 38 µm, the cerebral hemodynamic changes in the capillary or smaller arterioles may be not visualized and quantified in HVμDI. For example, a significant decrease in the cortical vascular density between young and aged WT groups was observed from the histological analysis (Figure 4d); however, the decrease between young and aged WT groups was not significant in the quantitative analysis from HVμDI (Figure 3e). This result may be attributed to that the histological analysis mainly visualize the capillaries (<10 µm) but the capillaries cannot be visualized by HVμDI. However, histological analysis cannot provide the hemodynamic information. Furthermore, the proposed HVμDI can be applied for both small and large animals but the maximum imaging depth (≈10–15 mm at 40 MHz) is another limitation. While this setting proves satisfactory for imaging entire hippocampal regions in small‐animal brains (e.g., the proposed imaging depth for rats is 12.5 mm^[^ ^16^ ^]^), it might be inadequate for capturing images of deeper brain regions. This includes pathological changes in the midbrain linked to Parkinson's disease, or when considering deeper brain areas in larger animals such as monkeys or piglets. Finally, in the current study, the AD model utilized was the 3xTg mice, which harbor three familial AD mutations (PS1_M146V_, tau_P301L_, and APP_Swe_) and display both amyloid and neurofibrillary tangle pathologies. The findings obtained from this model may not be directly extrapolated to most other AD mouse models that solely express mutated genes associated with Aβ metabolism and amyloid pathology. Exploring whether analogous vascular abnormalities manifest in AD models characterized solely by amyloidosis would be an intriguing avenue for future research. Further application of the proposed technique on different AD models remains imperative to comprehensively address this aspect.

## 4 Conclusion

In conclusion, a novel high‐spatiotemporal‐resolution ultrasound cerebrovascular imaging technique, namely HVμDI, was proposed in this study to visualize the cerebrovascular hemodynamics of mouse brains without using contrast agent. The proposed technique employs a morphology‐based enhancement algorithm and an adaptive filter, with a minimal detectable vessel diameter of 38 µm at a high temporal frame rate of 500 Hz. Several cerebrovascular properties—including the cerebrovascular density, vessel diameters, flow velocity, tortuosity, pulsatility, and reversal flow—were dynamically obtained and quantified from WT and AD mice through HVμDI. The cerebrovascular functions were altered by AD pathogenesis. Specifically, the cerebrovascular density, vessel diameters, flow velocity, pulsatility, and reversal flow were decreased in AD mice; however, the tortuosity was increased. The pulsatility and reversal flow were determined in the AD mouse model through ultrasound imaging, which enabled visualization under higher image resolutions than those in most studies that have used optical microscopy. The experimental data obtained in this study revealed that HVμDI yields high‐spatiotemporal‐resolution brain imaging in small animals. However, additional studies are required to explore the association between cerebrovascular hemodynamics and AD pathology.

## 5 Experimental Section

### Ultrafast High‐Frequency Ultrasound Imaging Configuration

Ultrafast HFUS image data were acquired from an ultrasound research platform (Vantage 256, Verasonics Inc., Kirkland, WA, USA) with a 256‐element 40‐MHz linear array transducer (Vevo MS550D, FujiFilm VisualSonics, Toronto, ON, Canada).^[^ ^52^ ^]^ Because of the small cranial window size of the murine brain (2 mm × 6 mm), only 128 elements in the middle of the transducer were excited. A three‐axis holder (VisualSonics Vevo integrated Rail System III, FujiFilm VisualSonics, Toronto, ON, Canada) was used to fix the transducer during imaging. After data acquisition, channel data were transferred to a personal computer via a PCI Express interface. IQ data were then obtained using the Vantage 256 built‐in delay‐and‐sum beamforming algorithm. All signals and images were processed using MATLAB (R2019b, MathWorks, Natick, MA, USA). Thereafter, 40‐MHz plane‐wave images were acquired with a pulse repetition frequency of 3500 Hz, and seven tilted plane wave angles (from −3° to +3° with a 1° increment) were employed, yielding an effective frame rate of 500 Hz. The acquired IQ data (*N_x_*, *N_z_*, and *N_t_* × *NA*, corresponding to the lateral, axial, and temporal dimensions and numbers of plane‐wave transmitting angles, respectively) were coherently compounded^[^ ^52^ ^]^ for the extraction and enhancement of cerebral flow data. In addition, the acquired IQ data were separated into four dimensions (*N_x_*, *N_z_*, *N_t_*, and *NA*) for vector flow imaging.

### Phantom Experiments

A straight microtube flow phantom was built using polyethylene with an inner diameter of 280 µm. Doppler test fluid (069‐DTF, CIRS Inc., Norfolk, VA, USA) was added to the microtube as blood‐mimicking fluid. A commercial syringe infusion pump (KDS‐100 780 100, KD Scientific Inc., Holliston, MA, USA) was used to generate a steady flow at various flow velocities from 1.8 to 14.4 mm s^−1^. The transducer was placed perpendicular to the flow microtube.

### Animal Models and Preparation

All animal experiments were conducted after the protocol had been approved by the Animal Care Committee of National Cheng Kung University, Taiwan (Institutional Animal Care and Use Committee Approval No. 105 199) and are reported in accordance with the Animal Research: Reporting in Vivo Experiments (ARRIVE) guidelines. C57BL/6 mice with no genetic modification divided into two age groups were selected as WT mice (4 months old, 8 mice; 11 months old, 8 mice). The weights of the mice were 24.6 ± 1.8 g (young group) and 28.8 ± 2.5 g (aged group). 3xTg mice (weight, 30.6 ± 2.0 g), which expressed three familial AD mutant genes (PS1_M146V_, tau_P301L_, and APP_Swe_) were purchased from Jackson Laboratory (Bar Harbor, ME, USA) and employed as AD mice (11 months old, 7 mice). All the mice were maintained in an environmentally controlled room (temperature: 23  ±  1  ^○^C, humidity: 55 ± 5%, 12/12‐h light/dark cycle beginning at 07:00) located in the National Laboratory Animal Center, Tainan, Taiwan. After the scalp was removed, a rotary tool (Ultimate 500, Nakanishi Inc., Kanuma, Tochigi, Japan) was used to create the cranial window. The body temperature of the mice was monitored and maintained at 37.0  ±  0.2 ^○^C to prevent animal instability and mortality during surgery or data acquisition. Acoustic coupling gel was applied for ultrasound wave transmission, which was first centrifuged (3000 rpm for 15 min) to prevent air bubbles from affecting the signal acquisition process.

### Singular Value Decomposition (SVD) Filtering

SVD filter was implemented using a block‐wise strategy to preliminarily extract blood signals.^[^ ^41^ ^]^ The compounded 3D IQ data (*N_x_*, *N_z_*, *and* *N_t_*) were spatially separated into several small blocks (*n_x_*, *n_z_*, and *N_t_*), and the block size was set to 64 (i.e., *n_x_* = *n_z_*  =  64). An overlap of 90% was set for the sliding block across the image to reduce the noise from the grid pattern artifacts. Subsequently, each small block was transformed into a Casorati matrix, *A* (*n_x_* × *n_z_*, *N_t_*). The matrix was then decomposed as

(1) $$A=U\Delta V^{H}$$

where *U* (*n_x_* × *n_z_*, *n_x_* × *n_z_*) and *V* (*N_t_*, *N_t_*) are left (spatial) and right (temporal) orthogonal matrices, H represents the Hermitian transpose, and Δ (*n_x_* × *n_z_*, *N_t_*) is a diagonal matrix with singular diagonal entries. The low‐ and high‐order singular thresholds (*T_L_* & *T_H_*) were adaptively determined according to the magnitude curve of singular values and the frequency curve derived from each singular vector *V*.^[^ ^40^ ^]^ Then, the threshold range δ was defined as *T_L_*: *T_H_*, and the filtered matrix *A_f_* was reconstructed as follows:

(2) $$A_{f}=U_{\delta }\;\Delta_{\delta }V_{\delta }^{H}$$

where *U*_δ_ represents *U*(:, δ), Δ_δ_ represents Δ(δ, δ), and *V*_δ_ represents *V*(:, δ). The global enhanced image was then reconstructed using the weighted averages of the filtered signals from different blocks. The enhanced image was obtained by summing the powers of the signals along the temporal direction. A cutoff frequency was adaptively determined through lag 1 autocorrelation on the *T_L_* th singular vector in the matrix *V*.

### Bowler‐Hat (BH) Transform and Hessian‐Based Vessel Enhancement (VE)

To achieve the effective visualization of cerebral vessels, BH transform^[^ ^53^ ^]^ and Hessian‐based VE^[^ ^54^ ^]^ were implemented. BH transform was first applied for morphological operations based on two structural elements (line and disk). For the disk‐shaped operation, the *ID* image can be expressed as

(3) $$\left\{\text{ID}\right\}=\left\{I\circ {\mathrm{SD}}_{r}:\forall r\in \left[1,\;r_{\max}\right]\right\}$$

where *I* is the input image (i.e., enhanced image after SVD filter), ◯ denotes the morphological opening operation, and *SD_r_* represents a bank of a disk‐shaped structural element of radius *r* ∈ [1,  *r_max_*]. For the line‐shaped operation, the *IL* image can be expressed as

(4) $$\left\{\mathrm{IL}\right\}=\left\{\max_{\varphi }\left(\left\{I\circ SL_{r,\varphi }:\forall \varphi \right\}\right):\forall r\in \left[1,\;r_{\max}\right]\right\}$$

where *SL*_*r*, *φ*_ denotes a bank of a line‐shaped structural element of radius *r* ∈ [1,  *r_max_*] and orientations *φ* ∈ [1,  *φ*_*max*_]; *r_max_* and *φ*_*max*_ were set as 30° and 180°. Subsequently, the enhanced image could be obtained using the maximum difference between Equations (3) and (4).

Hessian‐based VE was then performed to suppress the morphological imaging features resulting from the randomness of the noise in BH transform. Hessian‐based VE is based on the eigenvectors and eigenvalues of the Hessian matrix in the Cartesian coordinate (*x*, *y*):

(5) $$H_{\sigma }=\left[\begin{array}{cc} \frac{\partial^{2}}{\partial x^{2}}(I\left(x,y\right)*Gk\left(x,y,s\right) & \frac{\partial^{2}}{\partial x\partial y}(I\left(x,y\right)*Gk\left(x,y,s\right)\\ \frac{\partial^{2}}{\partial x\partial y}(I\left(x,y\right)*Gk\left(x,y,s\right) & \frac{\partial^{2}}{\partial y^{2}}(I\left(x,y\right)*Gk\left(x,y,s\right) \end{array}\right]\;$$

and

(6) $$Gk\left(x,y,s\right)=\frac{1}{2\pi \sigma^{2}}\;e^{\frac{x^{2}+y^{2}}{\sigma^{2}}}$$

where $H_{\sigma }$ is the Hessian matrix at scale σ (set from 1 to 2), *I* is the given input image, and * denotes the convolution operation. Then, a thresholding function based on the two eigenvalues (λ_1_ and λ_2_, assuming that |λ_2_| > |λ_1_|) of $H_{\sigma }$ ^[^ ^37^ ^]^ was applied to enhance the curvilinear structure as follows:

(7) $$\Delta_{\sigma }=\left\{\begin{array}{c} \;0,\;if\;\lambda_{2}>0\\ e^{-\frac{{\left(\lambda_{1}/\lambda_{2}\right)}^{2}}{2\beta^{2}}}\left(1-e^{-\frac{\lambda_{1}^{2}+\lambda_{2}^{2}}{2\alpha^{2}}}\right),otherwise \end{array}\;\right.\;$$

Parameters α and β are the parameters that control the sensitivity and were set to 2 and 1. The final enhanced image was obtained by taking the maximum value of Δ_σ_, and the vascular region was recognized by binarizing the final enhanced image (thresholding setting: dynamic range > 3 dB).

### Vector Flow Estimation Through a Multibeam Doppler Strategy

A fifth‐order Butterworth high‐pass filter with an adaptive determined cutoff frequency (obtained through SVD filtering) was temporally applied to the reshaped 4D IQ data (*N_x_*, *N_z_*, *N_t_*, *N_A_*), and the phase shift of the blood signals was calculated using lag 1 autocorrelation. The high‐pass filter and lag 1 autocorrelation for evaluating the phase shifts of the blood signals were operated individually at each tilted plane‐wave angle. Subsequently, vector‐flow processing based on a multibeam Doppler strategy was performed on the estimated phase shifts.^[^ ^39^, ^40^, ^55^, ^56^ ^]^ The axial velocity at a specific position (*x*′, *z*′, *t*′) from a specific plane‐wave transmitting angle θ_1_ was obtained using the following Doppler equation:

(8) $$v_{axial}\;\left(x^{\prime },z^{\prime },t^{\prime },\theta_{1}\right)=\frac{cFR}{4\pi f_{c}}\;\emptyset \left(x^{\prime },z^{\prime },t^{\prime },\theta_{1}\right)$$

where *c* is the speed of sound (1540 m ^−1^s), *FR* is the effective frame rate, *f_c_* is the operational frequency, and ∅(*x*′, *z*′, *t*′, θ_1_) is the phase shift. Then, the following linear equations can be formed:

(9) $$v\left(x^{\prime },z^{\prime },t^{\prime }\right)=v_{axial}\;\left(x^{\prime },z^{\prime },t^{\prime },\theta_{1}\right)/\cos\left(\theta_{D}\left(x^{\prime },z^{\prime },t^{\prime }\right)+\theta_{1}\right)$$

(10) $$v\left(x^{\prime },z^{\prime },t^{\prime }\right)=v_{axial}\;\left(x^{\prime },z^{\prime },t^{\prime },\theta_{2}\right)/\cos\left(\theta_{D}\left(x^{\prime },z^{\prime },t^{\prime }\right)+\theta_{2}\right)$$

(11) $$v\left(x^{\prime },z^{\prime },t^{\prime }\right)=v_{axial}\;\left(x^{\prime },z^{\prime },t^{\prime },\theta_{NA}\right)/\cos\left(\theta_{D}\left(x^{\prime },z^{\prime },t^{\prime }\right)+\theta_{NA}\right)$$

where *NA* is the plane‐wave angle and where *v*(*x*′, *z*′, *t*′) and θ_*D*_(*x*′,*z*′, *t*′) are the magnitude and direction of the velocity at the specific position, respectively, which were evaluated using the least‐squares fitting method. Theoretically, two linear equations (i.e., for two plane‐wave angles) are required to solve the two unknowns (magnitude and direction). The accuracy of the estimation increases with the number of tilted angles (≥ 3).

### Quantification

Cerebrovascular density was estimated on the basis of the final enhanced image (i.e., the image obtained after SVD filter, BH transform, and Hessian‐based VE). The cerebrovascular density was calculated as follows:

(12) $$Density=\frac{N_{pixels,\;BF}}{N_{pixels,\;ROI}}\times 100\%$$

where *N*_*pixels*, *BF*_ is the total number of blood signals within the region of interest (ROI) and where *N*_*pixels*, *ROI*_ is the total number of pixels in the ROI.

To estimate the cerebrovascular diameter, the final enhanced image was first binarized. The vascular binary mask was then skeletonized to determine the centerline of the vessel by using the MATLAB bwskel function. The vascular orientation was calculated by evaluating the morphological properties on the centerline by using the MATLAB regionprops function. For each pixel of the centerline, a segment normal to the vascular orientation of each pixel and centered on the centerline was selected to evaluate the cerebrovascular diameter through the full width at half maximum (FWHM). The minimal size of each segment was first set as the wavelength; if the FWHM could not be determined using the selected wavelength (i.e., less than two −3‐dB locations could be distinguished), the size incremented 10 µm.

The SOAM was then used to evaluate the cerebrovascular tortuosity. The horizontal and vertical velocities were smoothed using a 2D Gaussian smoothing filter with a standard deviation of 2. Subsequently, each pixel in the vascular region was first considered as an individual target, and its corresponding moving trajectory was then derived using frame rate and vector flow information. For any point *P_k_* in a specific flowing trajectory, the vectors *V*_*k* − 1_ and *V_k_* were defined as

(13) $$\begin{array}{c} V_{k-1}\;=\;\;P_{k}-P_{k-1}\\ V_{k}\;=\;P_{k+1}\;\;-P_{k} \end{array}$$

And the in‐plane angle *IP_k_* was obtained using dot product as follows:

(14) $${\mathrm{IP}}_{k}=\cos^{-1}\left(\left(\frac{V_{k-1}}{\left|V_{k-1}\right|}\right)\cdot \left(\frac{V_{k}}{\left|V_{k}\right|}\right)\right)$$

Finally, the SOAM could be used to evaluate the total tortuosity of the curve as follows:

(15) $$\mathrm{SOAM}=\frac{\sum_{k=1}^{n-3}\;{\mathrm{IP}}_{k}}{\sum_{k=1}^{n-1}\left|P_{k}-P_{k-1}\right|}$$

The hemodynamics of the cortical penetrating arterioles and ascending venules were assessed on the basis of the pulsatility index and reversal flow ratio. The penetrating arterioles and ascending venules were distinguished based on the average vertical flow direction in the temporal dimension (descending for penetrating arteries and ascending for ascending venules). The pulsatility index was calculated as the difference between the maximum velocity and minimal velocity divided by the averaging velocity in the temporal dimension.^[^ ^37^ ^]^ Reversal flow occurs was defined as the direction (both horizontal and vertical) of the flow changes in the temporal dimension more than two times (i.e., flowing backward and then forward). The reversal flow ratio was then calculated as the number of pixels that had reversal flow divided by the total number of pixels in the vascular regions.

### Flow Visualization

Dynamic flow rendering (Supplementary Videos S1–S3) and vector flow imaging (Supplementary Videos S4 and S5) were used to visualize the vector flow information in the brain. For dynamic flow rendering, data (including the B‐mode image, the enhanced image, and vector flow information) were interpolated threefold in the spatial dimension and twofold in the temporal dimension through spline interpolation. The enhanced image was used as a mask for the flow region. In addition, a randomized particle distribution was initialized in the flow region. The size of each particle was set to be proportional to the vector velocity in the corresponding voxel. The color of each particle was determined on the basis of the vector flow data on the HSV color map (with the hue denoting flow direction and the saturation denoting flow absolute velocity). The positions of these particles in the next temporal instants were dynamically updated on the basis of the vector flow data, and the particle size and color were changed on the basis of the flow information in the updated position. Particles were eliminated if their positions were beyond the flow region. The particle density was constantly controlled above 10%. Dynamic vector flow imaging was performed by replacing particles with arrows. The arrow lengths were determined on the basis of the vector velocity. A high spatial interpolation (fourfold) was performed for more favorable visualization in small regions (Supplementary Videos S4 and S5). The scale of the arrow length in Supplementary Video S5 was three times that of the scale in Supplementary Video S4 to enable more favorable visualization.

### Histological Analysis

Mice were anesthetized with Zoletil 50 (75 mg k^−1^g, i.p.; Virbac, Carros, France) and transcardially perfused with chilled phosphate buffered saline (PBS, Cat. #: 10 010 023, Thermo Fisher Scientific, Waltham, MA, USA). Their brains were removed, post‐fixed with 4% paraformaldehyde (Cat. #: 158 127, Sigma–Aldrich, St. Louis, MO, USA) prepared in 0.1 M phosphate buffer (Cat. #: P3619, Sigma–Aldrich) for 24 h, and dehydrated daily with gradually increasing concentrations (10, 20, 30, and 35%, twice per concentration) of sucrose (Cat. #: S9378, Sigma–Aldrich) solutions prepared in 0.1 M phosphate buffer (Cat. #: P3619, Sigma–Aldrich). The dehydrated brains were embedded in the cutting compound (Cat# 3 801 480, Leica Biosystems, Wetzlar, Hessen, Germany) and sliced into 25‐µm coronal sections using a cryostat (Model: CM1950, Leica Biosystems).

Double staining immunohistochemistry of CD31 and human Aβ were utilized to determine the vascular density and Aβ burden in the cortex and hippocampus of mice. The coronal brain sections containing these two regions (AP: −1.7 ∼ −2.3 mm from bregma) were mounted on silane coated slides (Cat. #: 511 614, Muto Pure Chemicals, Tokyo, Japan) and underwent antigen retrieval procedures using proteinase K (Cat. #: P2308, Sigma–Aldrich, 20 µg mL^−1^ dissolved in TE buffer containing 50 mm Tris Base, 1 mm EDTA, 5 mm CaCl_2_, and 0.5% Triton X‐100, pH 8.0) digestion for 40 min at 37 °C. Then, the brain sections were washed with PBS (Cat. #: 10 010 023, Thermo Fisher Scientific) containing 0.3% Triton X‐100 (Cat. #: X100, Sigma–Aldrich) (PBST), incubated with PBST containing 3% H_2_O_2_ (Cat. #: H1009, Sigma–Aldrich) for 20 min at room temperature, blocked with 3% normal goat serum (Cat. #: S26‐M, Sigma–Aldrich) prepared in PBST for 1 h at room temperature, and probed with primary antibodies against CD31 (1: 20 dilution, Cat. #: 550 274, BD Biosciences, Franklin Lakes, NJ, USA) for 16 h at room temperature. After washing out unbound antibodies, the brain sections were incubated with HRP‐conjugated goat anti‐rat IgG (1:200 dilution, Cat. #: 112‐035‐167, Jackson ImmunoResearch, West Grove, PA, USA) for 2 h at room temperature, followed by PBST washes, and incubated with the nickel‐enhanced chromogen, 3,3′‐Diaminobenzidine (Cat. #: D12384, Sigma–Aldrich). Then, the sections were further probed with antibodies against human Aβ (Cat. #: 800 712 and 803 004, BioLegend, San Diego, CA, USA; 1:250 dilution for each) for 16 h at room temperature, incubated with HRP‐conjugated goat anti‐mouse IgG (1:500 dilution; Cat. #: 115‐035‐166, Jackson ImmunoResearch) for 2 h at room temperature, and reacted with 3,3′‐Diaminobenzidine. Omissions of primary antibodies were used for detecting nonspecific binding in both staining procedures. After dehydration, the sections were mounted with the xylene‐based mounting medium (Cat. #: 3 801 730, Leica Biosystems), and the images were captured by an optical fluorescence microscope (Model: Axio Imager A1, Carl Zeiss, Oberkochen, Germany) equipped with a digital camera (Model: Axiocam 305 Color, Carl Zeiss). The vascular density, revealed as the area fraction of CD31‐immunoreavtive signals, in the region of interests was quantified using ImageJ software (v2.0.0‐rc‐69/1.52p, U.S. National Institutes of Health, Bethesda, MD, USA). The CD31‐immunoreavtive signals were separated from the double staining images using color deconvolution plugin of ImageJ software. A consistent background threshold was set and applied to all analyses and the over‐threshold pixels were counted as immunoreactive signals. The analyzers who counted the cells were blinded to the treatment. Three sections from each region of interest of each animal were determined, and the average was presented as a single data point.

### Statistics

All the experimental data were presented as means ± standard deviations (SDs). Variations among the animal groups were assessed using a one‐way analysis of variance. The significance level was set at *p* <0.05.

## Conflict of Interest

The authors declare no conflict of interest.

## Author Contributions

All authors conceived and designed the research. H.H. performed the ultrasound experiments and processed the data. H.H. and C.C.H. analyzed the data and wrote the manuscript. Y.M.K. and C.C.H. provided the materials to perform the experiments. P.L. H. and S.F.T. analyzed the histological data. Y.H.C., D.Q.C., G.X.X, and C.C. performed the phantom and animal experiments.

## Supporting information

Supporting Information

Supplemental Video 1

Supplemental Video 2

Supplemental Video 3

Supplemental Video 4

Supplemental Video 5

## Data Availability

The data that support the findings of this study are available from the corresponding author upon reasonable request.
