# Supplementary material for: High‐Spatiotemporal‐Resolution Ultrasound Flow Imaging to Determine Cerebrovascular Hemodynamics in Alzheimer's Disease Mice Model
Source: Adv Sci (Weinh). 2023 Nov 14;10(35):2302345. doi: 10.1002/advs.202302345 (PMC10724386; doi:10.1002/advs.202302345)
Supplement: Supplementary file 1 — Supporting Information [file ADVS-10-2302345-s003.pdf]

ADVANCED SCIENCE
Open Access

Supporting Information

for *Adv. Sci.*, DOI 10.1002/advs.202302345

High-Spatiotemporal-Resolution Ultrasound Flow Imaging to Determine Cerebrovascular Hemodynamics in Alzheimer's Disease Mice Model

*Hsin Huang, Pei-Ling Hsu, Sheng-Feng Tsai, Yi-Hsiang Chuang, De-Quan Chen, Guo-Xuan Xu, Chien Chen, Yu-Min Kuo and Chih-Chung Huang**

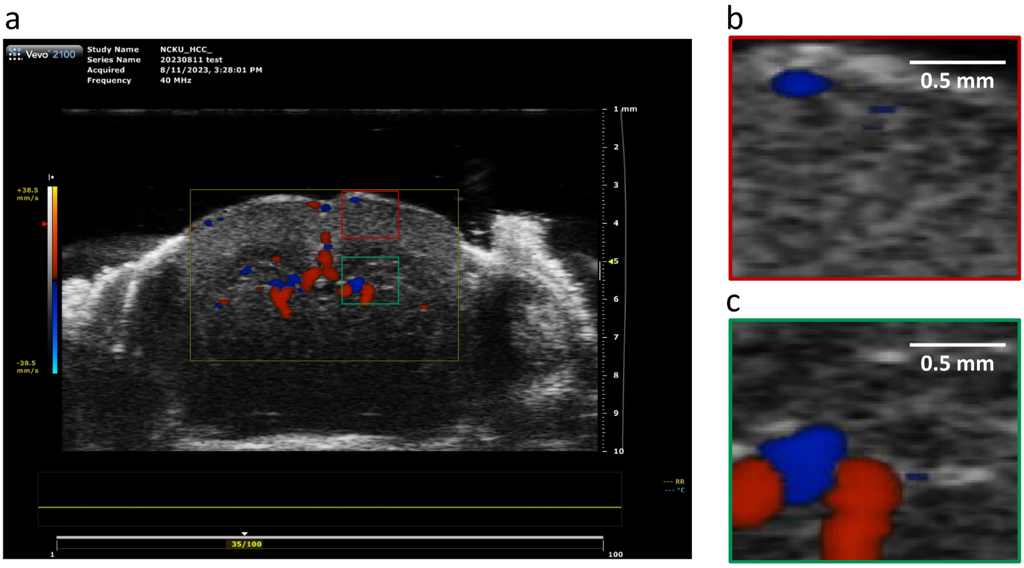

Figure S1: Cerebral vasculature images of a living mouse (young WT) brain from a commercial high-frequency ultrasound imaging system (VisualSonics Vevo 2100). a, Color Doppler image of mice brain. The operational frequency was set to 40 MHz and the "slow flow" mode was selected for the cerebral vasculature image. Only few blood flows were obtained from the larger vessels. **b and c,** Zoomed-in images in cortical (**b**) and hippocampal (**c**) regions (red and green windows in **a**), respectively. Again, there were few flow signals were detected in cortical region, and only larger cerebral flows in hippocampal region were obtained. Besides, the commercial system cannot provide the vector flow information.
